# Identification of Pre-Spike Network in Patients with Mesial Temporal Lobe Epilepsy

**DOI:** 10.3389/fneur.2014.00222

**Published:** 2014-10-28

**Authors:** Nahla L. Faizo, Hana Burianová, Marcus Gray, Julia Hocking, Graham Galloway, David Reutens

**Affiliations:** ^1^Centre for Advanced Imaging, University of Queensland, Brisbane, QLD, Australia; ^2^ARC Centre of Excellence in Cognition and its Disorders, Macquarie University, Sydney, NSW, Australia; ^3^School of Psychology and Counseling, Queensland University of Technology, Brisbane, QLD, Australia; ^4^Royal Brisbane and Women’s Hospital, Brisbane, QLD, Australia

**Keywords:** interictal spikes, hippocampus, mesial temporal lobe epilepsy, EEG-fMRI, functional connectivity, network

## Abstract

**Background:** Seizures and interictal spikes in mesial temporal lobe epilepsy (MTLE) affect a network of brain regions rather than a single epileptic focus. Simultaneous electroencephalography and functional magnetic resonance imaging (EEG-fMRI) studies have demonstrated a functional network in which hemodynamic changes are time-locked to spikes. However, whether this reflects the propagation of neuronal activity from a focus, or conversely the activation of a network linked to spike generation remains unknown. The functional connectivity (FC) changes prior to spikes may provide information about the connectivity changes that lead to the generation of spikes. We used EEG-fMRI to investigate FC changes immediately prior to the appearance of interictal spikes on EEG in patients with MTLE.

**Methods/principal findings:** Fifteen patients with MTLE underwent continuous EEG-fMRI during rest. Spikes were identified on EEG and three 10 s epochs were defined relative to spike onset: spike (0–10 s), pre-spike (−10 to 0 s), and rest (−20 to −10 s, with no previous spikes in the preceding 45s). Significant spike-related activation in the hippocampus ipsilateral to the seizure focus was found compared to the pre-spike and rest epochs. The peak voxel within the hippocampus ipsilateral to the seizure focus was used as a seed region for FC analysis in the three conditions. A significant change in FC patterns was observed before the appearance of electrographic spikes. Specifically, there was significant loss of coherence between both hippocampi during the pre-spike period compared to spike and rest states.

**Conclusion/significance:** In keeping with previous findings of abnormal inter-hemispheric hippocampal connectivity in MTLE, our findings specifically link reduced connectivity to the period immediately before spikes. This brief decoupling is consistent with a deficit in mutual (inter-hemispheric) hippocampal inhibition that may predispose to spike generation.

## Introduction

Mesial temporal lobe epilepsy (MTLE) is the most common symptomatic focal epilepsy and is frequently associated with hippocampal sclerosis (HS), i.e., neuronal cell loss and gliosis of the hippocampus (1, 2). While HS has been understood to represent a focal neuro-pathological alteration linked to the generation of seizures (i.e., the epileptogenic focus) (3), not all patients become seizure free after surgical resection of the hippocampus (4). Hence, the concept of the epileptogenic focus has been revised to incorporate the involvement of an “epileptogenic network” of brain regions, in which the hippocampus is a key component (5).

Epileptogenic networks have been explored via single photon emission computed tomography (SPECT) (6), positron emission tomography (PET) (7), and simultaneous electroencephalography (EEG) and functional magnetic resonance imaging (EEG-fMRI) (8). Of these, EEG-fMRI has the potential to be the most informative, as it is able to provide highly spatially resolved three-dimensional maps of brain activation (fMRI), which can be linked to interictal electrical discharges seen on EEG. EEG-fMRI studies in patients with MTLE have demonstrated widespread activation and deactivation in temporal lobe structures, particularly in the hippocampus ipsilateral to scalp recorded interictal spikes, as well as in extra-temporal regions (9, 10). Perhaps more importantly, EEG-fMRI findings have also demonstrated hemodynamic alterations that occur immediately prior to interictal spikes (11, 12). These pre-spike BOLD changes were reported by Jacobs et al. (13) to be more focal than spike-triggered alterations reported by Kobayashi et al. (8) and Salek-Haddadi et al. (14), suggesting that hemodynamic alterations preceding interictal spikes may provide better localization of regions involved in spike generation (8, 13, 14).

A common way to identify functional brain networks is to assess functional connectivity (FC) between spatially separated regions. FC measures the degree of covariance between the activity in a specific brain region and other areas across the whole brain. In MTLE, decreased FC in ipsilateral mesial temporal lobe networks and increased contralateral compensatory connectivity during the interictal state have been reported (15, 16). Delineation of FC patterns related to interictal spikes may be useful in shedding light on the mechanisms that underlie these changes, and potentially MTLE seizures. Although the exact physiologic relationship between interictal spikes and seizures are not fully understood (17, 18), there is a growing evidence that the neural network involved in generating interictal spikes is a reliable estimator of the network that generates seizures (19–21). The aim of this study was to use EEG-fMRI to investigate FC changes immediately prior to the appearance of interictal spikes on EEG in patients with MTLE.

## Materials and Methods

### Participants

Fifteen patients (9 females, mean age: 38 years; 6 males, mean age: 42 years) with MTLE (10 left and 5 right lateralized) and 15 age-matched healthy controls participated in the study. Patients were recruited from the Royal Brisbane and Women’s Hospital Epilepsy clinic, whereas healthy participants were recruited via the University of Queensland Human Research volunteer scheme. All patients underwent comprehensive clinical assessment and the diagnosis of MTLE was based on the following: (a) seizure semiology consistent with MTLE; (b) interictal spikes confirmed during in-patient video-EEG monitoring performed within the last year, and (c) MRI scan consistent with a temporal lobe focus (no lesion or ipsilateral HS). Patient exclusion criteria included absence of interictal spikes during monitoring, recurrent unprovoked seizures, and the presence of metal implants. Patients’ clinical details and spike distributions are summarized in Table 1. Only one patient had been free of seizures for 6 months and recurrent seizures occurred in the remainder. All EEG-fMRI recordings were acquired during the interictal state. Healthy controls were screened for current or previous brain injury, neurological, or psychiatric disorders. All participants provided written informed consent prior to enrollment, and the study was approved by the Human Research Ethics Committee (HREC) at the Royal Brisbane Women’s Hospital (RBWH) and the Centre for Advanced Imaging, the University of Queensland.

**Table 1:** **Summary of the patients’ clinical details and spike distribution**.

Patients	Lateralization of epilepsy	Age of epilepsy onset	Duration of the disease (years)	Clinical MRI	Total number of spikes across 6 runs	AEDs
1	R	21	12	HS	47	Levetiracetam, gabapentin, clobazam
2	L	20	25	N	None	Levetiracetam
3	R	14	7	N	None	Levetiracetam, clobazam, valproate
4	R	21	26	HS	52	Levetiracetam, carbamazepine, valproate
5	R	21	2	N	None	Lamotrigine, carbamazepine, valproate
6	L	30	25	N	50	Levetiracetam, lamotrigine
7	L	17	6	N	24	Lamotrigine
8	R	16	14	HS	None	Levetiracetam, oxacarbazepine, clobazam
9	L	20	24	N	49	Levetiracetam, lamotrigine, phenytoin
10	L	17	3	N	35	Pregabalin, cabamazepine
11	L	25	7	N	32	Lamotrigine, oxacarbazepine, topiramate
12	L	23	21	N	54	Levetiracetam, lacosamide, valproate
13	R	35	2	N	37	Lacosamide
14	L	4	55	N	32	Carbamazepine, phenytoin, clonazepam
15	L	25	8	N	34	Carbamazepine, levetiracetam, lamotrigine, valproate

*R, right; L, left; HS, hippocampal sclerosis; N, normal; none, no spikes have been identified during EEG-fMRI; AEDs, anti-epileptic drugs*.

### Procedure

The study was conducted at the Centre for Advanced Imaging, the University of Queensland. An MRI compatible 64-channel electrode cap was positioned on patients’ heads according to the international 10:20 system and prepared with a conductive non-abrasive gel (chloride 10%). All electrodes, including the ground (AFz) and reference electrodes (FCz) impedances, were below 5 kΩ. One additional electrode recorded ECG from the chest. Patients then underwent a 40-min simultaneous EEG-fMRI recording, having been instructed to remain still, awake, and relaxed with their eyes closed. Healthy control participants underwent only resting state fMRI without the EEG recording.

### EEG data acquisition and preprocessing

Electroencephalography was acquired with an MR-compatible Brain Products EEG System (Brain Products, Gilching, Germany), using a 64-channels cap with silver silver/chloride (Ag/AgCl) electrodes. EEG data were recorded using Brain Vision Recorder software version 1.20.0001 (Brainproducts Co., Munich, Germany). After recording, EEG datasets were preprocessed using EEGLAB software (22). Gradient artifacts introduced by MRI scanning were corrected with the Artifact Slice Template Removal (FASTR) algorithm (23, 24). Low pass (70 Hz), high pass (1 Hz), and notch (50–60 Hz) filtering were then used to remove frequency movement artifacts. An optimal basis set was formed to define the variations in the pulse artifact and create a template, which was then subtracted from the EEG data. Residual artifacts were removed using independent component analysis (ICA). An expert neurologist then reviewed the preprocessed EEG records to identify interictal spikes. Three out of the 15 patients did not show any spikes throughout the recording, and the EEG of one other patient contained movement artifacts. These data were not included in further analysis.

### fMRI data acquisition and preprocessing

Structural and functional MR data were acquired using a 3 T Siemens Magnetom Trio scanner, with a 12-channel head coil. fMRI-BOLD weighted images with full brain coverage were acquired with a single-shot gradient-echo planar image sequence (36 slices, TR = 2500 ms, TE = 30 ms, flip angle = 90°, matrix = 64 × 64, 3.3 mm isotropic voxels). T1-weighted (MP-RAGE) anatomical images were acquired (192 slices, TR = 1900 ms, TE = 2.13 ms, flip angle = 9°, matrix = 192 × 256 × 256, 0.9 mm isotropic voxels). EEG-fMRI data were collected in six runs, with each EPI run lasting 5:05 min, and the anatomical images 4:35 min.

MRI preprocessing was conducted using SPM8 (Wellcome Trust Centre for Neuroimaging, London, UK), in Matlab (Mathworks, Sherborne, MA, USA) (http://www.fil.ion.ucl.ac.uk/spm/software/spm8/). Functional images were slice time corrected, realigned, and normalized via the SPM8 Segment routine prior to spatial smoothing with an 8 mm FWHM isotropic Gaussian kernel.

### fMRI analysis

Functional magnetic resonance imaging analysis was conducted in four steps, using *Partial Least Square* (PLS) software (25, 26). First, event-related analysis was used to identify activation in mesial temporal lobe, and, in particular, in the hippocampus ipsilateral to the seizure focus. Second, we examined the time course of activity within the hippocampal region. Third, we examined the FC of the peak voxel in this cluster to delineate large-scale networks during the spike, pre-spike, and rest periods. Finally, we tested whether the FC maps from the previous analysis were correlated with seizure recency, i.e., time from the last seizure. The three 10 s periods were defined relative to spike onset on EEG: spike (0–10 s), pre-spike (−10 to 0 s), and rest (i.e., baseline) (−20 to −10 s, with no previous spikes in the preceding 45 s). This time window was chosen because the hemodynamic response function returns to baseline 25 s after a single burst of neural activity (i.e., the interictal spike). Our study was designed to examine short-term changes in connectivity, and was based on previous findings that pre-spike BOLD signal alterations are evident up to 9 s before interictal spikes (13, 27). On this basis, we selected the interval between 25 s after a spike and 10 s before the next spike as baseline. A total of 186 spike onsets were included in the analysis. Images from patients with right TLE were flipped along the antero–posterior axis, so that in all patients the seizure focus was on the left. Therefore, all results were expressed as ipsilateral or contralateral, referring to the spikes recognized on the EEG.

Partial Least Square is a multivariate tool that enables delineation of distributed brain regions in relation to task demands (task PLS), behavioral performance (behavior PLS), or activity in a given seed region (seed PLS). Briefly, PLS uses singular value decomposition (SVD) of a single matrix that contains all participants’ data to identify latent variables (LVs) that explain the covariance in the data. Each LV consists of three components: singular image of brain saliences (the brain image that best reflects the correlation of the task or behavior changes across conditions), design saliences (a set of weights that indicate the relationship between brain activity in a singular brain image and each of the assigned conditions), and singular value (the amount of covariance captured by the LV). For each LV in each condition, brain scores are calculated by multiplying each voxel’s salience by the normalized BOLD signal value in the voxel, and summing across all brain voxels for each subject. Conceptually, brain scores represent the weighted average of the contribution each voxel makes to the specific pattern of connectivity. The statistical assessment is determined using a permutation test and bootstrap estimation of standard errors for the brain (voxel) saliences. Permutation tests assess the significance of the LV by resampling the singular value with participants being randomly reassigned (without replacement) to different conditions. Bootstrap resampling is independent of permutation, assessing by resampling the voxel saliences with replacement of subjects but maintained assignment of participants to conditions. Resampling with 100 bootstrap steps was satisfactory to estimate standard error of the voxel weights/saliences (bootstrap ratio or BSR) for each LV. Peak voxels above BSR of 3 (i.e., *p* < 0.002) were considered reliable. Corrections for multiple comparisons were not required because the extractions of brain saliences are calculated in a single mathematical step on the whole brain.

Event-related task PLS was conducted to identify spike-related activation. Then, the peak voxel time course within the activated region in the ipsilateral hippocampus was tested across the three epochs with four TRs per epoch, each TR being 2500 ms. PLS connectivity analysis was conducted using the peak voxel activated by spikes in the ipsilateral hippocampus as the seed voxel. BOLD signal intensities in that voxel were extracted and correlated with every other voxel in the brain in each condition across all subjects. The correlation of brain activity between the seed voxel and every other voxel in the brain across different conditions and subjects was calculated and stacked into a single combined matrix of correlations called the behavior matrix. The behavior matrix was then decomposed with SVD into a set of LVs that describe the network/regions (FC pattern) that correlated with the ipsilateral hippocampal activity in different conditions. Finally, to examine the relation between FC patterns in the three states (spike, pre-spike, and rest) and seizure recency, we conducted seed/behavior analysis by adding the time from last seizure (in weeks) as a variable in the subsequent PLS connectivity analysis. We were thus able to assess whether spike, pre-spike, or resting FC maps, defined in relation to the ipsilateral hippocampus, were related to interval from last seizure.

## Results

### Whole brain analysis

**Figure 1:**
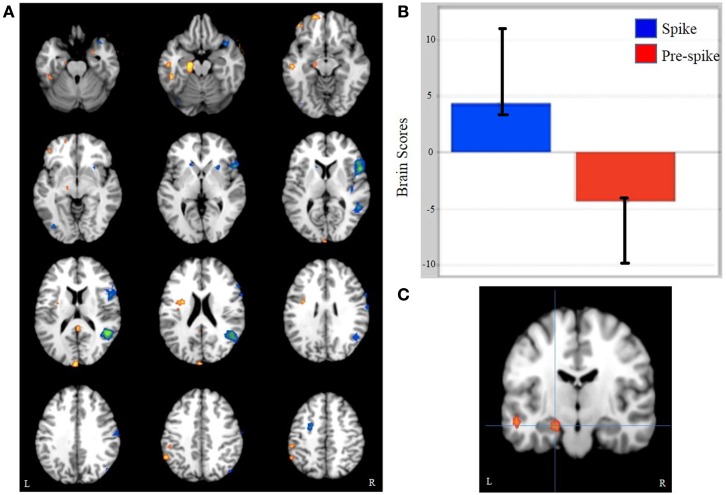
**Task PLS results**. **(A)** A pattern of whole brain activity in spikes versus pre-spike. **(B)** Brain scores related to the pattern seen in **(A)**. **(C)** L hippocampus activation cluster, from which the peak voxel was used for functional connectivity analysis.

Event-related task PLS analysis of spike, pre-spike, and rest states yielded significant activity in the ipsilateral mesial temporal structures. As hypothesized, spike-related activation was seen in the ipsilateral hippocampus (relative to pre-spike) and was accompanied by increased activity in the ipsilateral parahippocampal gyrus, middle temporal gyrus, precuneus, contralateral middle temporal gyrus, and insula (Figure 1; Table 2). Additionally, activity in the ipsilateral medial frontal gyrus and the right inferior and superior frontal gyri were decreased during interictal spikes, relative to the pre-spike period.

**Table 2:** **Whole brain analysis, spike versus pre-spike**.

Region	Side	Peak MNI coordinates			Ratio^a^
		*x*	*y*	*z*	
Positive correlations
HP, para HP, amygdale	L	−15	−15	−12	4.34
Middle temporal gyrus	R	69	6	−21	4.62
Precuneus	R	3	−42	69	4.11
	L	−3	−40	71	4.08
Middle temporal gyrus	L	−52	−20	−10	4.04
Insula	R	−30	−12	−18	3.49
Negative correlations
Inferior frontal gyrus	R	51	15	6	−6.99
Superior frontal gyrus	R	48	−48	15	−6.28
Medial frontal gyrus	L	−12	−18	66	−4.24

*HP, hippocampus; L, left; R, right; MNI, Montreal Neurological Institute; SE, standard error*.

*^a^Salience/SE ratio in bootstrap analysis*.

**Figure 2:**
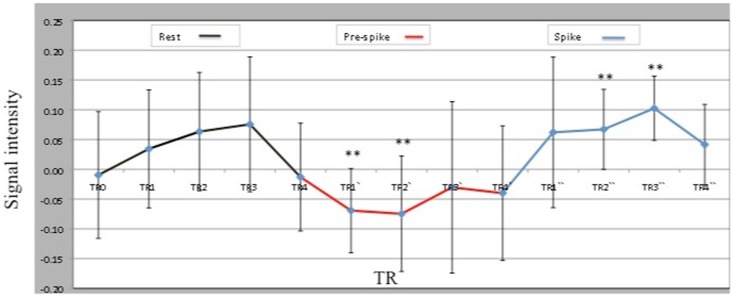
**Peak voxel (−21, −27, −12) BOLD signal intensities within the ipsilateral hippocampal activation across three conditions: rest, pre-spike, and spike**. TRs, TRs′, and TRs″ represent the 4TRs for rest, pre-spike, and spike, respectively. Each TR is 2.5 s.

Analysis of the time course and degree of activation in the peak voxel within the ipsilateral hippocampal cluster (MNI coordinates; −21, −27, −12) revealed a decrease in ipsilateral hippocampal activity during the 10 s pre-spike period when compared to rest and spike conditions (Figure 2). Paired *t*-tests showed that spike and pre-spike time courses differed significantly between TR1′, TR2′ during pre-spike and TR2″,TR3″ during spike (*p* = 0.002, *p* = 0.005, respectively).

### Functional connectivity analysis

**Figure 3:**
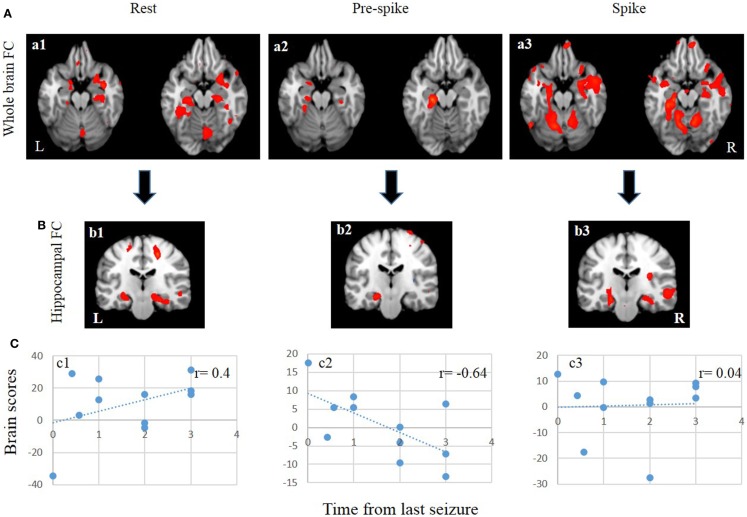
**FC and seed/behavior results**. **(A)** From left to right, patterns of whole brain FC during rest (a1), pre-spike (a2), and spike (a3). **(B)** From left to right, patterns of bilateral hippocampal FC during rest (b1), pre-spike (b2), and spike (b3). **(C)** From left to right, seed/behavior correlation between FC maps in a1 (c1), a2 (c2), and a3 (c3).

During the rest epoch, the ipsilateral hippocampus was functionally connected with the contralateral hippocampus, and the parahippocampal gyri, fusiform gyri, amygdala, and cerebellar cortex bilaterally (Figures 3Aa1,Bb1; Table 3). Activity in the ipsilateral hippocampus was also correlated with structures of the default mode network including the precuneus, bilateral superior frontal, medial temporal, and cingulate gyri. The strongest connectivity, however, was demonstrated with the contralateral hippocampus and the parahippocampal gyri, amygdala, and cerebellar cortices bilaterally.

**Table 3:** **Functional connectivity pattern during rest**.

Region	Side	Peak MNI coordinates			Ratio^a^
		*x*	*y*	*z*	
Para HP, amygdale	L	−21	−12	−15	47.09
HP, para HP, amygdale	R	24	−15	−4	13.5
Cerebellum	L	−24	−51	−9	29.32
	R	31	−53	−15	15.22
Fusiform	L	−36	−42	−14	4.51
	R	37	−40	−15	4.00
Precuneus	R	2	−72	47	4.03
	L	−2	−72	51	4.59
Cingulate gyrus	R	16	−29	42	4.007
	L	−21	−26	42	4.32
Superior frontal gyrus	R	30	51	51	7.68
	L	−5	19	58	3.48
Medial frontal gyrus	L	−20	−2	42	6.20
Medial temporal gyrus	R	50	−4	−20	6.85
	L	−50	−2	−23	6.08
Brainstem		0	−23	−23	6.85

*HP, hippocampus; L, left; R, right; MNI, Montreal Neurological Institute; SE, standard error*.

*^a^Salience/SE ratio in bootstrap analysis*.

During the pre-spike period, the ipsilateral hippocampus showed connectivity to the ipsilateral parahippocampal gyrus, bilateral cerebellar cortices, ipsilateral insula, bilateral lentiform nuclei, and contralateral caudate nucleus (Figures 3Aa2,Bb2; Table 4).

**Table 4:** **Functional connectivity pattern during pre-spike**.

Region	Side	Peak MNI coordinates			Ratio^a^
		*x*	*y*	*z*	
Para HP, amygdale	L	−18	−15	−11	16.92
Middle temporal gyrus	L	−61	−28	−11	5.04
Caudate	R	20	25	−10	7.95
Lentiform nucleus	L	−13	6	−13	7.2
	R	17	6	−11	8.12
Cingulate gyrus	L	−7	22	30	−9.2
	R	8	21	33	−6.05
Insula	L	−43	−15	−10	4.55
	R	44	−13	−10	4.55

*HP, hippocampus; L, left; R, right; MNI, Montreal Neurological Institute; SE, standard error*.

*^a^Salience/SE ratio in bootstrap analysis*.

At the time of spikes, the ipsilateral hippocampus showed a connectivity pattern similar to the pattern of connectivity during rest, except for increased connectivity to the contralateral insula (Figures 3Aa3,Bb3; Table 5). Also, in the spike epoch, negative correlations were observed with both superior frontal gyri. The main differences between pre-spike and spike conditions were that during pre-spike, the connectivity of the ipsilateral hippocampus to the contralateral hippocampus, both parahippocampal gyri and cerebellar cortex were significantly reduced, whereas negative correlation in activity was observed with insula, lentiform nuclei, and cingulate gyri bilaterally.

**Table 5:** **Functional connectivity pattern during spike**.

Region	Side	Peak MNI coordinates			Ratio^a^
		*x*	*y*	*z*	
Para HP, amygdale	L	−25	−15	−15	23.05
HP, para HP, amygdale	R	29	−15	−13	5.01
Cerebellum	L	−23	−53	−10	29.32
	R	31	−52	−15	15.22
Fusiform	R	38	−65	−3	4.60
Insula	R	44	−42	25	9.11
	L	−2	−72	51	4.59
Lentiform nucleus	L	−20	−15	−8	13.75
Red nucleus		0	−15	−7	6.05
Superior frontal gyrus	L	−18	21	58	−4.68
	R	24	20	58	−5.6
Middle frontal gyrus	L	−36	5	44	−6.72

*HP, hippocampus; L, left; R, right; MNI, Montreal Neurological Institute; SE, standard error*.

*^a^Salience/SE ratio in bootstrap analysis*.

Seed/behavior correlation analysis revealed similar maps to those seen in the previous FC analysis (Figure 3C). Importantly, this additional analysis showed that seizure recency was strongly correlated with the pre-spike (a negative correlation of *r* = −0.64) (Figure 3, c2) and rest conditions (a positive correlation *r* = 0.4) (Figure 3, c1), but not with the spike condition (Figure 3, c3).

## Discussion

We used EEG-fMRI to investigate FC changes immediately prior to the appearance of interictal spikes on EEG in patients with MTLE. Our findings showed spike-related activation in the ipsilateral hippocampus. In addition, we demonstrated the significantly reduced ipsilateral hippocampal activity, and the loss of bilateral hippocampal FC immediately before the appearance of electrographic spikes. Moreover, we showed that the pre-spike connectivity pattern is related to seizure recency, suggesting that the altered FC changes prior to spikes was influenced by the time from last seizure. Spike-related activation in the ipsilateral hippocampus is consistent with previous EEG-fMRI studies on patients with MTLE (8, 28, 29).

In the FC analysis, the most striking finding was the significant loss of connectivity between the hippocampi several seconds before the appearance of spikes on EEG. During rest and spiking, there was a coupled coherence between the two hippocampi. However, this coherence decreased dramatically a few seconds prior to the onset of interictal spikes and are in keeping with a role for altered inter-hippocampal interaction in the initiation of spikes.

The hippocampi are anatomically and functionally connected by the fornix (30), a major input and output pathway for the hippocampus (31, 32). Previously, it was thought that seizure and epileptiform discharges are initiated in one hippocampus and propagate to the contralateral hippocampus through the fornix. However, the short delay (20 ms) between activity in right and left hippocampi raises the possibility that the hippocampi are functionally synchronized (33). Studies of inter-hippocampal synchronization using intracranial EEG in animals and human beings have shown that normally, there is electrophysiological coherence between the hippocampi in the delta wave frequency range during wakefulness (0.5–2 Hz) (34, 35) and rapid eye movement sleep (36). Functional synchronization may involve the input that both hippocampi receive from each other via commissural fibers in the fornix. In animal models of MTLE, there is significant loss of synchronization at high frequencies between the hippocampi prior to the onset of epileptiform discharges (37). Our results support and translate these findings into human beings using EEG-fMRI FC analysis. We found that the loss of coherent synchronization between the two hippocampi occurred a few seconds before the appearance of interictal spikes.

Previous studies on animal models of focal epilepsy have shown hemodynamic changes prior to spikes (38, 39). These pre-spike changes have been related to early synchronization of a population of neurons before interictal discharges. In human beings, EEG-fMRI has also demonstrated early BOLD changes in the pre-spike period. Both positive and negative pre-spike BOLD changes have been described and have been found to be more focal than the spike-related BOLD signals. Correlation of early BOLD changes with findings from invasive EEG recording has revealed pre-spike synchronized neural discharges from areas exhibiting early BOLD changes (27). These pre-spike EEG discharges were observed on the intracranial EEG but not detected with scalp EEG.

Interictal inter-hemispheric hippocampal FC (40) has been investigated using resting state fMRI in MTLE. Decreased FC within the ipsilateral temporal lobe and between temporal lobe structures in both hemispheres has been reported. EEG-fMRI has been used to examine the relationship between connectivity and brain states related to interictal spikes. In these studies, reduced FC between the hippocampus ipsilateral to the seizure focus with the contralateral hippocampus has been reported in relation to interictal activity in patients with unilateral MTLE, when compared to controls (41). Pereira et al. (42) has demonstrated that healthy subjects exhibit high FC between the hippocampi, whereas in patients with MTLE, the basal connectivity between the hippocampi is disrupted. Our findings support and extend the knowledge from previous reports of reduced bilateral hippocampal activity. Specifically, we showed that the loss of connectivity between the hippocampi is linked to the pre-spike period. Our approach in defining different brain states (i.e., background, pre-spike, and spike) facilitated the identification of altered FC during the transition from rest to spike states. It remains to be determined whether these changes in FC are due principally to changes in firing patterns in the ipsilateral (abnormal) hippocampus, the contralateral hippocampus, or to a complex desynchronized pattern of firing in both hippocampi. It is possible that decreased connectivity reflects a reduction in inter-hemispheric inhibition from the contralateral hippocampus, which plays a role in the emergence of interictal spikes. Further research is needed to differentiate between these alternatives. Seizure recency influenced short-term connectivity patterns. The shorter the interval from the last seizure, the greater the recruitment of the pre-spike network, whereas the rest network was more strongly recruited with longer intervals from the last seizure.

This study and others have emphasized the usefulness of EEG-fMRI and FC in examining brain connectivity in disease, but conclusions from these studies should take into account their limitations. In our study, the possibility that not all interictal spikes were visible in scalp recorded EEG (43) may limit the accuracy and specificity of our analysis. Additionally, we report findings in a small sample of patients, which is likely to have reduced statistical power (44). Each subject was scanned only once, and the FC patterns were derived from the average of all pre-spike periods across all subjects. Each patient had a differing number of spikes, as reported in Table 1, and our estimates of FC were based on the average of all pre-spike periods available. The variability in connectivity across epochs and subjects is taken into account in the statistical inference insofar as significant voxels represent the consistent features of the connectivity maps. Furthermore, the large range of AEDs prescribed and the relatively low number of subjects precluded the analysis of the influence of specific drug classes on connectivity patterns. Finally, we concede there may be a degree of temporal blurring in examining connectivity time linked to interictal spikes in a dataset with a temporal resolution of 2.5 s. However, if it were possible to remove this effect, the focal pattern of connectivity that we observed during the pre-spike period might be expected to be even stronger.

To conclude, our main findings indicate that ipsilateral hippocampal activity and FC are reduced during the period immediately prior to the appearance of interictal spikes. These findings may provide insights about the patho-physiological state of mesial temporal lobe structures underlying the genesis of spikes.

## Conflict of Interest Statement

The authors declare that the research was conducted in the absence of any commercial or financial relationships that could be construed as a potential conflict of interest.
